# Supplementary material for: Statistical analysis plan for the Steppedwedge Cluster Randomized trial of Electronic Early Notification of sepsis in hospitalized ward patients (SCREEN)
Source: Trials. 2021 Nov 22;22:828. doi: 10.1186/s13063-021-05788-3 (PMC8607063; doi:10.1186/s13063-021-05788-3)
Supplement: Supplementary file 2 — Additional file 2. Statistical analysis plan [file 13063_2021_5788_MOESM2_ESM.docx]

**STATISTICAL ANALYSIS PLAN**

| ***Stepped-wedge Cluster Randomized Trial of Electronic Early Notification of Sepsis in Hospitalized Ward Patients***  ***(SCREEN Trial)***  Clinicaltrials.gov identifier: NCT04078594 |
| --- |

SAP Version: Date of Statistical Analysis Plan:

1.0 08-AUG-2021


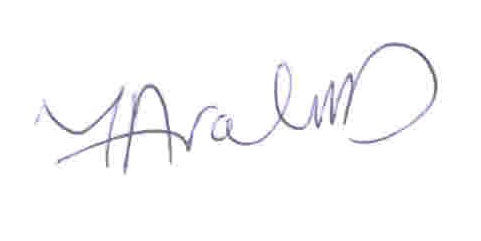


Principal Investigator: _____________________________

Dr. Yaseen Arabi


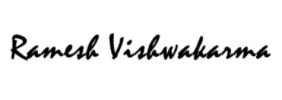


Statistician: _______________________

Dr. Ramesh Kumar Vishwakarma

**Table of Contents**

1. INTRODUCTION 4

1.1 Study design 4

1.2 Randomization 4

1.3 Trial population 5

1.3.1 Trial centres 5

1.4 Study objectives 6

1.5 Primary objective 6

1.6 Secondary outcomes 6

2. POPULATIONS FOR ANALYSIS 7

2.1 Intention-to-treat cohort 7

2.2 Alert cohort 8

2.3 Levels of confidence and p values 8

2.4 Multiple testing 8

2.5 Protocol deviations 9

2.6 Integrity of data 9

3. HYPOTHESES AND DECISION RULES 9

3.1 Statistical hypotheses 9

3.2 Statistical decision rules 9

3.2.1 Testing significance for primary endpoint 9

4. INTERIM ANALYSIS 10

5. STATISTICAL METHODOLOGY 10

5.1 Sample size justification 10

5.2 Data handling 11

5.2.1 Methods for handling missing data 11

5.3 Patient disposition 12

5.3.1 Demographics and baseline disease characteristics 12

5.4 Efficacy analyses 13

5.4.1 Primary analysis 13

5.4.2 Model checking 13

5.4.3 Other Secondary Efficacy Endpoints 15

5.4.4 Analysis of secondary outcomes 18

5.4.5 Sensitivity analyses 18

6. CHANGES TO PLANNED ANALYSES FROM PROTOCOL 19

7. PROGRAMMING CONSIDERATIONS 19

7.1 Statistical Software 19

**LIST OF ABBREVIATIONS AND GLOSSARY OF TERMS**

| **Abbreviation** | **Term** |
| --- | --- |
| CONSORT | Consolidated Standards of Reporting Trials |
| CCRT | Critical Care Response Team |
| CI | Confidence interval |
| ICU | ICU Intensive care unit |
| ITT | Intention to treat |
| PP | Per protocol |
| qSOFA | Quick Sequential Organ Failure Assessment |
| SAP | Statistical analysis plan |
| SOFA | Sequential Organ Failure Assessment |
| SWCRT | Stepped-wedge cluster randomised trial |

# INTRODUCTION

The Stepped-wedge Cluster Randomized Trial of Electronic Early Notification of Sepsis in Hospitalized Ward Patients (SCREEN) study is designed as a stepped-wedge cluster randomized trial to evaluate the effect of screening for sepsis using an electronic sepsis alert versus no alert in hospitalized patients admitted to wards with active sepsis alert system compared with wards with no active (masked) sepsis alert system on all-cause in-hospital mortality by 90 days. In keeping with present practice, and taking into considerations traits of trial design and reporting specific to non-pharmacologic interventions. The purpose of the Statistical Analysis Plan (SAP) is to provide a framework that addresses the protocol objectives in a statistically rigorous fashion, with minimized bias or analytical deficiencies. Specifically, this plan has the following purpose:

To prospectively (a priori) outline the types of analyses and data presentations that will addresses the study objectives outlined in the protocol, and to explain in detail how the data will be handled and analysed, adhering to commonly accepted standards and practices.[1]

## Study design

This study is designed as a stepped-wedge cluster randomized trial. This design is is used for evaluation of interventions related to service delivery. A stepped wedge cluster randomised controlled design allows to sequentially deliver the interventions to all trial clusters over a number of time period (Table 1).

## Randomization

We present a glossary of terms in line with the CONSORT extension for stepped-wedge cluster randomized trials.[1] The cluster refers to the unit of randomization, which is the hospital ward, and we will refer to it in the rest of the document as “ward”. A list of ward-level and patient-level eligibility criteria is outlined in the study protocol.[2, 3] After a baseline period of 2 months, the intervention is implemented in a new sequence of five new randomly selected wards, until it is eventually implemented in all 9 sequences (45 wards) (Figure 1). Therefore, the 9 sequences are randomized over a total of 10 periods, the first being the baseline. Wards will be randomised to one of 9 sequences according to a computer-generated non-stratified concealed list. The randomization list will be maintained with a research coordinator who is not involved in this trial, and the ward allocation remained concealed from the research and clinical teams throughout the study, and will be revealed for a given sequence only 1 month before the implementation of the intervention to allow training. (**Figure 1**)

**Figure 1:** Stepped-wedge cluster randomized trial design.

## Trial population

### Trial centres

### The study is conducted in the 5 Ministry of National Guard Health Affairs (MNGHA) hospitals which share the same Electronic Medical Record (EMR) system (BESTCare, South Korea). These hospitals are King Abdulaziz Medical City- Riyadh, King Abdulaziz Medical City- Jeddah, and Prince Mohammed Bin Abdul Aziz Hospital – AlMadinah, King Abdulaziz Hospital - Al Ahsa, and Imam Abdulrahman Al Faisal Hospital – Dammam. All MNGHA hospitals have an integrated Electronic Medical Record (EMR) system, BESTCare, which has been implemented as a joint venture with the Seoul National University Bundang Hospital, South Korea. Its critical applications include the clinical documentation, computerized physician order entry, clinical decision support system, and clinical data warehouse. The system is also interfaced with the hospital's vital signs measurement devices.

**Table 1:** Total number of eligible wards in the MNGHA hospitals.

| Center | Number of wards |
| --- | --- |
| KAMC-RIYADH | 25 wards |
| KAMC-JEDDAH | 9 wards |
| KAH- ALAHSA | 6 wards |
| PMBAH- MADINAH | 4 wards |
| IABFH - DAMMAM | 2 wards |
| **Total** | **46*** |

*Any two of the wards with homogeneous respect to certain attributes will be combined for purposes of randomization.

## Study objectives

The aim of the trial is to determine whether active sepsis alert system, compared with no active (masked) sepsis alert system, is associated with a reduction in 90 day all-cause mortality rate in ward patients.

## Primary objective

All-cause in-hospital mortality within 90 days

## Secondary outcomes

- - - Hospital length of stay (LOS), censored at 90 days
    - Transfer to ICU within 90 days (ITT cohort) and 14 days of alert (alert cohort)
    - ICU-free days in the first 90 days (ITT cohort and alert cohort)
    - Critical Care Response team (CCRT) activation within 90 days (ITT cohort) and 14 days of alert (alert cohort)
    - Cardiac arrest within 90 days (ITT cohort) and 14 days of alert (alert cohort)
    - The need for mechanical ventilation, vasopressor therapy, incident renal replacement therapy within 90 days (ITT cohort) and 14 days of alert (alert cohort)
    - Organ dysfunction during hospitalization up to 90 days, assessed by electronic SOFA [4]
  - Balancing measures
    - Antibiotic-free days within 90 days (ITT cohort and alert cohort)
    - The acquisition of multidrug resistant organisms within 90 days (ITT cohort and alert cohort)
    - Clostridium difficile infection within 90 days (ITT cohort and alert cohort

# POPULATIONS FOR ANALYSIS

## Intention-to-treat cohort

We will report patient flow according to CONSORT flowchart for stepped wedge cluster randomized trial by allocated sequence and period[1][1] (Figure 1).[1] The intention-to-treat (ITT) cohort includes all eligible patients admitted to the eligible wards. The ITT analysis also implies that patients in the ITT cohort in the wards belonging to a particular period will be analyzed in accordance with their planned randomization regardless of what happens during the trial. For example, if a ward is planned to have the intervention during a given period and for technical reasons that alert system is not operational, patients recruited in that ward during that period will be analyzed as receiving active alert. Although it is not anticipated that there will be wards that cross-over their study cohort (i.e., change from alert to non-alert, or vice versa), any such instances will be documented. Patients who are transferred from one ward to another will be counted as part of the first ward. The primary analysis will be based on this population.

## Alert cohort

This cohort represents the subset of ITT patients who had the alert whether in the intervention wards or the control wards.

## Levels of confidence and p values

Statistical tests and their confidence intervals (CIs) will be calculated with two sided. The statistical significance level set will be at the 5% level. Between-cohort comparisons will be calculated and presented with 95% CIs wherever possible.

## Multiple testing

To adjust for multiple testing for secondary outcomes and subgroup analyses, we will use the False Discovery Rate (FDR). In this procedure all hypothesis tests will be sorted in an descending order based on their calculated p-value. All hypothesis tests below an index K will be rejected where K calculated as follows:

$$K=max\left\{ i:p\left( i \right)\leq\frac{i}{m}.q \right\}$$

*where* i =m, …, 1, m is the total number of tested hypotheses; q = 0.05.

The multiplicity testing adjustment will also be done on confidence intervals by constructing 1-K*q/m CI for each selected parameter.[5]

## Protocol deviations

We will document protocol deviations, if they occur, including failure to implement the intervention in a given ward, or wrong implementation of the intervention in a ward assigned to the control group. These deviations will be documented on the CONSORT flow diagram. The data from such wards will be analyzed according to the ITT principle.

## Integrity of data

All outcome data will be independently double entered. All inconsistencies between data entries will be investigated and any discrepancies discussed with, and resolved by, the principal investigator. The integrity of the data will be monitored regularly, with scrutiny of data files for omissions and errors. Range and sense checks will be performed on all variables prior to commencing statistical analyses.

# HYPOTHESES AND DECISION RULES

## Statistical hypotheses

Primary hypothesis to be tested:

The study hypothesis is that the implementation of sepsis alert system will result reducing in 90 days all-cause in-hospital mortality compared to no alert.

## Statistical decision rules

### Testing significance for primary endpoint

We will report risk difference and 95% confidence intervals for all-cause in-hospital mortality by day 90. All tests will be two-sided with a significance level of 0.05.

# INTERIM ANALYSIS

There will be no interim analysis due to the nature of implementation and the final analysis will be performed after all follow-up data have been collected.

# STATISTICAL METHODOLOGY

For the SCREEN trial, the analysis will be implemented under the principle of intension-to-treat and the guiding principles of the ICH E9 for the analysis of randomized trials. The stepped wedge design is essentially a matched design with before and after comparisons for each unit of randomization.[1] In this case, a unit is a ward where each ward is randomized to a particular starting point.

Raw data will be processed in accordance with the best practices for raw data management to identify any inaccuracies or incompleteness in advance of the statistical analysis. In order to accomplish this task, all interval variables will be checked and summarized in terms of maximum and minimum values. Minimum and maximum values will be checked and compared against the nominal maximum and minimum value of each variable, and variables with implausible values will be flagged. All variables will be summarized and reported for the study using descriptive statistics. Interval variables will be summarized and reported in terms of n, mean and median, standard deviation, Q1 and Q3. Categorical variables will be summarized and reported in terms of frequency distribution. All demographic and clinical variables would be summarized between study cohorts at baseline by wards and overall.

## Sample size justification

The sample size for this stepped-wedge cluster-randomized design is calculated for 45 clusters with 10 periods (including one baseline period) using Power Analysis and Sample Size (PASS) software (PASS 15 Power Analysis and Sample Size Software (2017). NCSS, LLC. Kaysville, Utah, USA, ncss.com/software/pass). After a baseline period, 5 new clusters (wards) switch from the control group to the intervention group at the beginning of each subsequent periods. Using historical data obtained from the development domain of the EMR) for ward patients admitted from 01 July 2018 to 30 June 2019, we calculated a baseline in-hospital mortality rate by day 90 of 3.13%. Based on the same dataset, 18.3% of eligible ward patients had the an alert based on qSOFA criteria with an in-hospital mortality of 8.16% compared to 2% in the no-qSOFA alert patients. For sample size calculations, we made the following assumptions: A) the impact of intervention on mortality occurs only in patients who have the alert, B) Only half of patients with the alert have sepsis, C) 90% of deaths among the patients with the alert occurred among septic patients, D) early intervention resulting from the alert will reduce the in-hospital mortality by 50%, i.e. from 8.16% to 4.08% in patients with sepsis and would lead to an overall change in in-hospital mortality for the whole cohort from 3.13% to 2.46%, yielding a relative risk of 0.79, D) 80% power using two-sided Wald Z-Test and significance level of 5% and, E) an intra-cluster correlation (ICC, a measure of the relatedness of cluster) of 0.22 as estimated from the same retrospective electronic database. As the primary analysis would be adjusted for random effect to account for the correlation between patients within the same cluster, we used the estimation variance (sigma²), which is calculated from responses (P1, P2), as the within-cluster variance (sigma w²) as suggested by Hussey and Hughes (2007) and Hemming and Girling (2014).[6, 7] As such, a reduction of in-mortality in hospital by 90 days by 0.67% (from 3.13% to 2.46%) requires a total sample size of 65250 subjects (average of 1450 subjects per cluster with an average of 145 subjects per cluster per period). With all five hospitals combined, this is expected to require 20 months (2 months per period). There is no planned interim analysis.

## Data handling

### Methods for handling missing data

We do not expect to have missing observations in the variables required for the primary analysis. Given the nature of the study, missing observations are expected, for example not all patients will have all laboratory tests and therapies within the narrow time windows defined the in the protocol. Variables used in descriptive analysis will not be imputed. In some of the secondary and sensitivity analyses, imputation will be used. Because Glasgow outcome score is not documented for patients with normal neurologic status, missing observations will be assigned a normal value for purposes of the model adjustment. Other variables used in model adjustment model will be assessed and characterized in terms of their pattern (i.e., Missing Completely at Random, Missing at Random, Missing Not at Random). For Missing Completely at Random data, all analyses will be based on list-wise deletion approach where observation will complete values will be only considered for analysis. For variables with values Missing at Random, multiple imputation techniques will be utilized to impute the missing values as suggested by Rubin’s.[8] And for variables with values Missing Not at Random, a pattern‐mixture model technique will be used to impute the missing values.[9]

## Patient disposition

The flow of patients will be displayed in accordance with CONSORT (Consolidated Standards of Reporting Trials) 2010 guidelines for reporting randomized trials and the CONSORT extension for stepped-wedge cluster randomized trials.[1, 10, 11]. This description will include information about eligibility criteria and follow-up losses at both cluster and patient levels.

### Demographics and baseline disease characteristics

#### Baseline characteristics

Baseline characteristics will be presented for the ITT and alert cohorts (Table S1) including age, sex, source of admission, admitting ward, comorbidities (extracted based on ICD-10AM), Charlson comorbidity index, source of infection (pneumonia, urinary tract infection, skin and soft tissue infection, intra-abdominal infection, other infections, and no clear source, extracted based on ICD-10AM) and dialysis. We will also report vital signs (systolic blood pressure, diastolic blood pressure, heart rate, temperature, and respiratory rate) as well as laboratory parameters (lactate, white blood cells, bilirubin, creatinine) and whether culture of blood, respiratory, urine or other body fluids (pleural, ascitic, CSF, joint) are obtained and whether treatments (intravenous fluids and antibiotics) are received at baseline.

## Efficacy analyses

### Primary analysis

The primary outcome of all-cause in-hospital mortality by day 90 will be compared between the intervention group and control group at the individual level with a generalized linear mixed model with a binary distribution using with jack-knife method to estimates standard errors to account for grouping within clusters and by incorporating a log-link function to estimate the relative risk as a measure of effect.[6, 12]. We will include two levels of random effects to account for nested clustering within wards and periods and two levels of fixed effects; hospitals and COVID-19 ward status in addition to intervention. Results will be expressed as relative risk with 95% confidence interval (CI). In case any models fail to converge, we will report ORs from mixed effect logistic regression.

### Model checking

Parameter estimates may be non-robust to the failure of the assumed distribution of the random effects, therefore diagnostic checking of the residuals will be performed. The over dispersion of parameters will also be assessed by calculating the ratio of the Pearson chi-square statistic and its degrees of freedom. If this ratio exceeds 1, indicates that the variability in data has not been properly modeled and that there is residual over dispersion due to misspecification of conditional distribution. In case of no event reported in a cluster for a particular period of time, a firth correction will be used in generalized linear mixed model to avoid issue of quasi-separation. In case of Poisson regression, If data deviates from assumption of equidispersion ( average = variance). The Pearson Chi-Square / DF value should be roughly 1.0. A value greater than 1.0 indicates the data are over-dispersed, i.e., the variance is greater than the mean. In general, a value greater than 2.0 requires remedial action. Therefore, we will be using robust variance estimators to estimate robust standard errors. When the data really are from a Poisson process (no violation of equidispersion assumption) , the Poisson SE may be preferred as it will generally provide more statistical power in this situation; the P-values and CIs will be smaller. The model will be selected as the best model with a unique covariance structure that produces the lowest Bayesian Information Criterion (BIC) value. The covariance structures that will be considered in the model are: first order of autocorrelation covariance structure, unstructured covariance structure, Toeplitz covariance structure and variance component structure (VC).We will use Satterthwaite method to adjust for denominator degree of freedom for tests of the fixed effects. The random coefficients will be modeled using G-side random effects and obtain the subject-specific estimates by defining the appropriate variance-covariance structure. The overdispersion of parameters will also be assessed by calculating the ratio of the Pearson chi-square statistic and its degrees of freedom. If this ratio exceeds 1, it indicates that the variability in data has not been properly modeled and that there is residual overdispersion due to misspecification of the conditional distribution. In case of no event reported in a cluster for a particular period, a firth correction will be used in the generalized linear mixed model to avoid the issue of quasi-separation. The Newton-Raphson optimization technique with ridging option might be used to help with the convergence of the model: Results will be expressed as relative risk with 95% confidence interval (CI). The primary outcome will be also analyzed similarly in the alert cohort. In case any models fail to converge, we will report ORs from mixed effect logistic regression for the primary outcome.

### Other Secondary Efficacy Endpoints

#### Processes measures

The data will be summarized by number and percentages for each ward by hospital and overall for following measures:

- - - - - Post-alert lactate level

Percentage of patients with lactate reported 12 hours if not reported in the 12 hours before alert

Highest value reported in the 12 hours after the alert

- - - - - Post-alert blood culture:

Percentage of patients with blood culture ordered in 12 hours if not performed in the 12 hours before alert

- - - - - Post-alert respiratory, urine and body fluid cultures

Percentage of patients with respiratory, urine and body fluid cultures ordered in 12 hours if not performed in the 12 hours before alert

Intravenous fluid administered in 12 hours after alert (yes, no)

- - - - - Post-alert antibiotics

Percentage of patients who are not on antibiotics in the 12 hours before alert and had new antibiotic administered within 3 and 12 hours of alert

Percentage of patients who are on antibiotics in the 12 hours before alert and had new antibiotic administered within 3 and 12 hours of alert

- - - - - Post-alert systolic blood pressure: lowest value in the 12 hours after the alert.
        - Post-alert diastolic blood pressure: lowest value in the 12 hours after the alert
        - Post-alert heart rate: highest value in the 12 hours after the alert
        - Post-alert respiratory rate: highest value respiratory in the 12 hours after the alert

#### Physiological parameters

The physiological parameters at baseline will be summarized for ITT population and pre-alert (Alert population)

- Systolic and diastolic blood pressure: lowest values in the first 12 hours of check in to the ward (ITT population) and in the 12 hours before the alert
- Heart rate and respiratory rate: highest values in the first 12 hours of check in to the ward (ITT population) and in the 12 hours before the alert
- Temperature: highest value and lowest value in the first 12 hours of check in to the ward (ITT population) and in the 12 hours before the alert
- Lactate level: highest lactate 12 hours before check in to the ward to 12 hours of check-in to the ward (ITT population) and highest value in the 12 hours before the alert (alert population).
- White blood cell count, bilirubin, and creatinine: highest values in the 12 hours before check-in to the ward to 12 hours after check-in to the ward (ITT population) and highest value in the 12 hours before alert (alert population)
- Blood, respiratory, urine and body fluid cultures: percentage of patients with cultures ordered in the 12 hours before check-in to the ward to 12 hours after check-in to the ward (ITT population) and in the 12 hours before alert (alert population)
- Intravenous fluid (NS, ½NS, D5NS, D5½NS, LR, D5LR, albumin 5%, 20%) given in the 12 hours before check-in to the ward to 12 hours after check-in to the ward (ITT population) and in the 12 hours before the alert (alert population)
- Antibiotics: percentage of patients on antibiotics in the 12 hours before check-in to the ward to 12 hours after check-in to the ward (ITT population) and in the 12 hours before alert (alert population)

#### Balancing / safety measures

The data will be summarized using number, median and inter quartile range (IQR) for each ward by hospital and overall for following measures:

- - - Antibiotic-free days within 90 days (ITT cohort and alert cohort)
    - The acquisition of multidrug resistant organisms within 90 days (ITT cohort and alert cohort)
    - Clostridium difficile infection within 90 days (ITT cohort and alert cohort)

#### Outcome measures

- - - Hospital length of stay (LOS), censored at 90 days: the hospital length of stay will be defined as “number of days patient stayed at the hospital” and can be calculated by considering the difference between date of discharge to date of check-in. The no-of days patient stayed in the hospital will be analysed using a mixed effect Poisson model. We will include two levels of random effects to account for nested clustering within wards and periods and two levels of fixed effects; hospitals and COVID-19 ward status in addition to the intervention.
    - Transfer to ICU within 90 days (ITT cohort): this outcome will be defined as “number of days patient transferred to ICU” and can be calculated by the proportion of patients transferred to ICU from date of check-in and will be analysed in similar manner as done for primary outcome.
    - ICU free days (ITT cohort): the outcome will be defined as time between date of ICU admission to date of check-in and will be compared in the similar manner as done for hospital length of stay outcome.
    - Critical Care Response team (CCRT) activation within 90 days (ITT cohort): this outcome will be defined as “CCRT activation within 90 days”. The proportion of patients with CCRT activation in the ICU will be analyzed in similar manner as done for primary outcome.
    - Cardiac arrest within 90 days (ITT cohort): this outcome will be defined as “Cardiac arrest within 90 days from the checkin”. The proportion of patients with Cardiac arrest in the ICU will be analyzed in similar manner as done for primary outcome.
    - The need for mechanical ventilation, vasopressor therapy, incident renal replacement therapy within 90 days (ITT cohort) : these outcomes will be analyzed in similar manner as done for primary outcome.

### Analysis of secondary outcomes

Categorical outcomes including ICU admission, CCRT activation, cardiac arrest, the need for mechanical ventilation, vasopressor therapy, incident renal replacement therapy, acquisition of multidrug-resistant organisms and *Clostridium difficile* infection will be compared between the intervention and the control group, in a similar model to the one used in the analysis primary outcome. We will include two levels of random effects to account for nested clustering within wards and periods and two levels of fixed effects; hospitals and COVID-19 ward status in addition to the intervention. Results will be expressed as relative risk with 95% CI. In case any models fail to converge, we will report ORs from mixed-effect logistic regression.

Continuous outcomes including hospital length of stay (LOS), ICU-free days, and antibiotic-free days will be compared using a mixed effect Poisson model (Table S3). We will include two levels of random effects to account for nested clustering within wards and periods and two levels of fixed effects; hospitals and COVID-19 ward status in addition to the intervention. The model will be selected as best model with a unique covariance structure that produces the lowest Bayesian Information Criterion (BIC) value. The covariance structures that will be considered in the model are: first order of autocorrelation covariance structure, unstructured covariance structure, Toeplitz covariance structure and variance component structure (VC).” The random coefficients will be modeled using G-side random effects and obtain the subject-specific estimates by defining the appropriate variance-covariance structure. The results will be expressed as beta estimates with 95% CI.

###

### Sensitivity analyses

To address the concern about contamination, we will conduct a sensitivity analysis excluding all patients in the control group in the 90 days before crossing over to the intervention group. Because there are fewer than 50 clusters, we will conduct a sensitivity analysis using a small sample correction with a Kenward-Roger method.[6, 13, 14] We will conduct a sensitivity analysis adjusting for following covariates: type of wards (medical, surgical, oncology and mixed), age, baseline systolic blood pressure, baseline respiratory rate, GCS and Charlson comorbidity index. For the later analysis, we will use imputation for missing variables as outlined below. In addition, we will conduct also a complete case sensitivity analysis. We will conduct a sensitivity analysis excluding the periods in which wards are assigned as COVID-19 ward.

We will analyze the primary outcome of all-cause hospital mortality by day 90 across predefined subgroups using the same model of the primary analysis. The predefined subgroups include age=< 65 years and >65 years, patients with documented infection source (including ICD-10AU for pneumonia, urinary tract infection, skin and soft tissue infection, intra-abdominal infection or other infections) and patients with no documented infection or infection source, patients admitted to medical, surgical, oncology and mixed wards, alert within 48 hours of admission and after 48 hours of admission and patients admitted to COVID-19 and non-COVID-19 wards.

# CHANGES TO PLANNED ANALYSES FROM PROTOCOL

Not applicable.

# PROGRAMMING CONSIDERATIONS

## Statistical Software

All analyses will be performed using SAS version 9.4.

**References**

1. Hemming K, Taljaard M, McKenzie JE, Hooper R, Copas A, Thompson JA, Dixon-Woods M, Aldcroft A, Doussau A, Grayling M *et al*: **Reporting of stepped wedge cluster randomised trials: extension of the CONSORT 2010 statement with explanation and elaboration**. *BMJ* 2018, **363**:k1614.

2. Arabi YM, Saawi AA, Zahrani MA, Khathaami AA, AlHazme RH, Mutrafy AA, Qarni AA, Shouabi AA, Qasim EA, Abdukahil SA *et al*: **Electronic early notification of sepsis in hospitalized ward patients: a study protocol for a stepped-wedge cluster randomized controlled trial**. *medRxiv* 2021:2021.2005.2020.21257511.

3. Arabi YM, Alsaawi A, Al Zahrani M, Al Khathaami AM, AlHazme RH, Al Mutrafy A, Al Qarni A, Al Shouabi A, Al Qasim E, Abdukahil SA *et al*: **Electronic early notification of sepsis in hospitalized ward patients: a study protocol for a stepped-wedge cluster randomized controlled trial**. *Trials* 2021, **22**(1):695.

4. Control CfD, Prevention: **Hospital toolkit for adult sepsis surveillance**. *Atlanta, US Department of Health and Human Services* 2018.

5. Benjamini Y, Yekutieli D, Edwards D, Shaffer JP, Tamhane AC, Westfall PH, Holland B: **False Discovery Rate: Adjusted Multiple Confidence Intervals for Selected Parameters [with Comments, Rejoinder]**. *Journal of the American Statistical Association* 2005, **100**(469):71-93.

6. Hussey MA, Hughes JP: **Design and analysis of stepped wedge cluster randomized trials**. *Contemp Clin Trials* 2007, **28**(2):182-191.

7. Hemming K, Haines TP, Chilton PJ, Girling AJ, Lilford RJ: **The stepped wedge cluster randomised trial: rationale, design, analysis, and reporting**. *BMJ* 2015, **350**:h391.

8. *LITTLE, RJA , and D B RUBIN (1987) Statistical Analysis with Missing Data New York: John Wiley & Sons*.

9. Thijs H, Molenberghs G, Michiels B, Verbeke G, Curran D: **Strategies to fit pattern-mixture models**. *Biostatistics* 2002, **3**(2):245-265.

10. Moher D, Hopewell S, Schulz KF, Montori V, Gotzsche PC, Devereaux PJ, Elbourne D, Egger M, Altman DG: **CONSORT 2010 explanation and elaboration: updated guidelines for reporting parallel group randomised trials**. *BMJ* 2010, **340**:c869.

11. Schulz KF, Altman DG, Moher D, Group C: **CONSORT 2010 statement: updated guidelines for reporting parallel group randomised trials**. *BMJ* 2010, **340**:c332.

12. Shakeshaft A, Doran C, Petrie D, Breen C, Havard A, Abudeen A, Harwood E, Clifford A, D'Este C, Gilmour S *et al*: **The effectiveness of community action in reducing risky alcohol consumption and harm: a cluster randomised controlled trial**. *PLoS Med* 2014, **11**(3):e1001617.

13. Thompson JA, Hemming K, Forbes A, Fielding K, Hayes R: **Comparison of small-sample standard-error corrections for generalised estimating equations in stepped wedge cluster randomised trials with a binary outcome: A simulation study**. *Stat Methods Med Res* 2021, **30**(2):425-439.

14. Kenward MG, Roger JH: **Small sample inference for fixed effects from restricted maximum likelihood**. *Biometrics* 1997, **53**(3):983-997.
